# Isolation and identification of an isomeric sildenafil analogue as an adulterant in an instant coffee premix

**DOI:** 10.1080/20961790.2020.1829375

**Published:** 2020-11-11

**Authors:** Ahmad Yusri Mohd Yusop, Linda Xiao, Shanlin Fu

**Affiliations:** a Centre for Forensic Science, University of Technology Sydney, Ultimo, Australia; b Pharmacy Enforcement Division, Ministry of Health, Selangor, Malaysia

**Keywords:** Forensic sciences, food adulteration, non-targeted screening, phosphodiesterase 5 inhibitors, structural isomer, suspected-target screening

## Abstract

The proliferation of adulterated health foods and beverages in the market demands a comprehensive analytical strategy to identify the adulterants, particularly those of isomeric phosphodiesterase 5 (PDE5) inhibitors. An instant coffee premix (ICP) purchased from an online retailer was flagged for suspected adulteration through PDE5 inhibition assay. The ICP was then analysed using suspected-target and non-targeted screenings of a liquid chromatography-quadrupole time-of-flight mass spectrometry. Based on these findings, a PDE5 inhibitor initially assigned as compound X was isolated from the ICP by employing a liquid chromatography-diode array detection before its structural elucidation with liquid chromatography-ultraviolet (LC-UV) spectroscopy and nuclear magnetic resonance (NMR) spectroscopy. The suspected-target screening matched the protonated molecule ([M + H]^+^) precursor ion of compound X at *m/z* 499.2310 with two suspected analytes that are structural isomers of one another. The fragmentation patterns of compound X were comparable to those analogues in the dithiocarbodenafil group through the non-targeted screening. These findings, complemented by the LC-UV and NMR spectroscopy data, together with the chromatographic separation of related structural isomers, conclude the identity of compound X. To our best knowledge, this is the first study to report the presence of 3,5-dimethylpiperazinyl-dithiodesmethylcarbodenafil in an ICP sample.

**Key points**
- The herbal-based male sexual performance products’ lucrative market has instigated their rampant adulteration, particularly with PDE5 inhibitors.
- The adulterated products may also contain analogues of the approved PDE5 inhibitors, which usually passed into the market undetected as they are not included in the routine targeted screening procedure.
- The present study detected, isolated, and identified an isomeric sildenafil analogue from an instant coffee premix sample using rapid qualitative assay and comprehensive analytical analysis.
- This paper highlighted the applicability of the established strategies for routine casework, particularly in a forensic drug testing laboratory.

## Introduction

Herbal-based consumable products are widely perceived as healthy and safe compared to modern medicines [1]. Catchphrases such as all-natural, certified organic, and chemical-free are usually associated with these products to attract consumers. Moreover, the dispersal of misleading information, prominently through social networking media, along with aggressive Internet marketing strategies, has frequently deceived consumers [2]. Among the most prevalent are health foods and beverages that advertise to enhance male sexual performance [3]. These products often stated on their labels to supposedly made up of herbal aphrodisiacs such as *Panax ginseng*, *Eurycoma longifolia*, and *Lepidium meyenii*, to name a few [4].

Regrettably, this lucrative market entices a widespread adulteration, particularly with synthetic erectile dysfunction drugs, namely phosphodiesterase 5 (PDE5) inhibitors [5]. Worse, these products usually contain analogues of the approved drugs, viz. sildenafil, vardenafil, and tadalafil, which frequently passed through undetected as they are not included in the routine targeted screening procedure applied by forensic drug testing laboratories [6]. An analogue of PDE5 inhibitors is often synthesised by minor modifications to the parent structure of the approved drugs; thus, altering their physical and chemical properties [7]. Furthermore, some of these analogues are structural isomers of one another, making their identification a challenging task [8].

Clinical studies have shown that the approved PDE5 inhibitors may produce common side effects such as headache, flushing, dyspepsia, and abnormal vision [9]. Besides, they may also cause severe drug-drug interactions in patients on nitrates or α-blockers [10]. Contrarily, structural modifications on the unapproved analogues may impact their absorption, distribution, metabolism, and excretion, which could result in unpredictable potency and side-effects [11]. For example, a sildenafil analogue, namely propoxyphenyl-thiohydroxyhomosildenafil, is 10-fold more potent in inhibiting PDE5 enzyme compared to sildenafil [12]. Therefore, at the same dose, the analogue is more likely to cause severe side effects compared to sildenafil. Another analogue, acetildenafil, has been reported to trigger ataxia, a side effect that was never documented for PDE5 inhibitors before [7]. This adulteration trend raises serious concerns about food safety and public health, as consumers are often unaware of the risks associated with consuming such products [13]. A fatality case associated with a sildenafil analogue, i.e. desmethylcarbodenafil [14], highlights the need for a comprehensive analytical strategy that may reveal the presence of PDE5 inhibitors, particularly those of the unapproved analogues.

In this study, an instant coffee premix (ICP) was submitted to a comprehensive analytical procedure with a pre-screening using PDE5 inhibition assay, where it was flagged for suspected adulteration. The ICP was then analysed to detect specific PDE5 inhibitors through suspected-target and non-targeted screenings. A suspected PDE5 inhibitor, initially assigned as compound X, was isolated from the ICP to determine its identity.

## Materials and methods

### Chemicals and reagents

An ICP (SPL005) promoted as a male sexual performance product was purchased from an established online retailer based in Malaysia. Certified reference materials (CRMs) of dithiodesmethylcarbodenafil, sildenafil impurity 12 (3,5-dimethylpiperazinyl-dithiodesmethylcarbodenafil), and sildenafil impurity 18 (dithiopropylcarbodenafil) were purchased from TLC Pharmaceutical Standards Ltd. (Aurora, Ontario, Canada). Liquid chromatography mass spectrometry (LC-MS) grade methanol and acetonitrile were purchased from Chem-Supply Pty Ltd. (Gillman, SA, Australia) while Sigma Aldrich Pty Ltd. (Castle Hill, NSW, Australia) supplied the LC-MS grade formic acid, analytical grade ammonium formate, and deuterated chloroform (CDCl_3_). Ultrapure water (18.2 MΩ-cm) was collected from a Sartorius arium^®^ pro ultrapure water system (Goettingen, Germany) while LECO Australia Pty Ltd. (Castle Hill) provided the QuEChERS extraction salt (EN 15662).

### Screening of food products

Health foods and beverages marketed with claims to enhance male sexual performance were pre-screened using PDE5 inhibition assay [15]. In brief, the bioactivity-based assay utilised a fluorescence polarisation technique to screen PDE5 inhibitors in foods and beverages, by competing with fluorescein-labelled cyclic-3′,5′-guanosine monophosphate (PDE5 substrate) to bind to PDE5 enzyme. Suspected products were flagged by their average PDE5 inhibition above the threshold value of the blank ICP matrix.

The suspected products (high priority samples) were then analysed to detect specific PDE5 inhibitors, through suspected-target and non-targeted screenings, with an Agilent Technologies (Santa Clara, CA, USA) 1290 Infinity II liquid chromatography (LC) system coupled to an Agilent Technologies 6510 quadrupole time-of-flight mass spectrometer (QTOF-MS) as described previously [16]. Briefly, the suspected-target screening employed a personal compound database and library (PCDL), comprising 95 PDE5 inhibitors and their analogues, providing extended coverage of known analytes without the need for CRMs. A suspected analyte was detected by comparing the accurate mass of the precursor ion to the theoretical ones in the library. The observed product ions were then compared to the common fragmentation patterns of target analytes, confirming the suspected analyte. Isomeric analytes belonging to the same group of PDE5 inhibitors, however, could not be distinguished as they shared the same common fragmentation patterns. The non-targeted screening was concomitantly engaged to flag potentially novel PDE5 inhibitors analogues based on the common fragmentation patterns of target analytes; where the top-down and bottom-up approaches were established to screen visible and non-visible chromatographic peak, respectively.

The chromatographic separation was carried out using a reverse-phase high-performance LC column from Merck KGaA (Darmstadt, Germany) Chromolith^®^ High-Resolution RP-18 end-capped (100 × 4.6 mm, 2.0 µm) with 10 mmol/L ammonium formate in ultrapure water (solvent A) and acetonitrile (solvent B) as the binary mobile phase system. Both solvents were acidified with 0.1% v/v formic acid. The gradient elution was set as follows: 5% B for 0–1 min, 5%–25% B for 1–2 min, 25%–50% B for 2–32 min, 50%–95% B for 32–33 min, and 95% B for 33–34 min at 0.4 mL/min. The elution was immediately returned to the initial gradient at 34.01 min for 6 min at 1 mL/min with post-run equilibration maintained at 0.4 mL/min, 5 min before the next injection. The QTOF-MS was operated in positive electrospray ionisation mode using a data-dependent acquisition.

SPL005, in the form of an ICP, is promoted to improve erectile function, prolong an erection, and prevent premature ejaculation, among others. The online advertisement also included a certificate of analysis stating that the ICP is free from adulterants such as sildenafil, vardenafil, and tadalafil. The ingredients listed on the ICP sachet were as follows: *Eurycoma longifolia*, *Lepidium meyenii*, arabica coffee, goat’s milk, creamer, and brown sugar.

### LC-UV analysis

The ultraviolet (UV) spectra were recorded on-line during the chromatographic run from 200–400 nm, with an Agilent Technologies 1290 Infinity LC coupled to an Agilent Technologies 1290 Infinity diode array detection (DAD), using the same chromatographic conditions as the screening of food products, with the UV signal monitored at 356 nm.

### Nuclear magnetic resonance (NMR) spectroscopy

The isolated compound X was dissolved in CDCl_3_ with ^1^H and ^13^C NMR spectra recorded using an Agilent Technologies 500 MHz NMR spectrometer coupled to an Agilent Technologies 7510-AS automated NMR sample changer at room temperature. The acquisition was performed at 499.86 MHz within 1 024 scans for ^1^H NMR and 125.70 MHz within 10 000 scans for ^13^C NMR. All the chemical shifts were reported in δ (ppm); measured relative to CDCl_3_ (^1^H δ = 7.26, ^13^C δ = 77.0). The coupling constants (*J* values) were expressed in Hertz (Hz).

### Standard solution preparation

Each CRM was prepared into a stock solution of 1 mg/mL in methanol and stored at 4 °C in the dark. A working solution was freshly prepared at 3 µg/mL for each analysis from the stock solution by further dilution in methanol.

### Sample preparation

One-third of SPL005 contents, i.e. 8.5 g (25.5 g in total per ICP sachet), were extracted using a modified QuEChERS procedure described previously [17]. For each extraction, 100 mg of the sample was dissolved in 5 mL of acetonitrile and methanol (1:1, v/v), sequentially *via* 1-min vortexing, 20-min sonication, and 5-min centrifugation at 2500 × *g*. The resulting mixture was then transferred into a tube prefilled with QuEChERS salt for extraction, by vortexing for 1 min, followed by centrifuging for 5 min at 2500 × *g*. The solutions from each extraction (85 extractions in total) were filtered and combined into a round bottom flask. The volume of the filtrate was subsequently reduced to 5 mL using a rotary evaporator.

### Isolation of compound X

Compound X was isolated from SPL005 through an Agilent Technologies 1290 Infinity LC system fitted with an end-capped high-performance LC column: Nucleoshell RP 18 (100 × 4.6 mm, 2.7 µm) from Macherey-Nagel GmbH & Co. KG (Duren, Germany). The column compartment temperature was maintained at 20 °C with an injection volume of 20 µL. The mobile phases, consisting of solvent A (10 mmol/L ammonium formate in ultrapure water) and solvent B (acetonitrile), were acidified with 0.1% v/v of formic acid. A shorter gradient elution programme was devised specifically for the isolation of compound X at 0.4 mL/min as follows: 5% B for 0–1 min, 5%–45% B for 1–2 min, 45%–65% B for 2–8 min, 65%–95% B for 8–9 min, and 95% B for 9–10 min. The system was immediately returned to the initial gradient with post-run equilibration maintained for 3 min before the next injection.

The fraction of compound X was collected following a DAD at 356 nm using an Agilent Technologies 1290 Infinity DAD. The procedure was repeated a number of times to obtain enough compound X for the LC-UV and NMR spectroscopy analysis. The collected fractions were then combined and placed under a gentle stream of nitrogen gas to remove the residual solvents.

### Data analysis

The qualitative and quantitative data of the LC-QTOF-MS, LC-DAD, and LC-UV analyses were processed through an Agilent Technologies Mass Hunter workstation software version B.07.00, Mass Hunter qualitative analysis software version B.07.00, and PCDL manager software version B.04.00. A Bruker (Billerica, MA, USA) TopSpin software version 4.0.6 was applied to analyse the NMR data.

## Results and discussion

### Screening of SPL005

**Figure 1.**
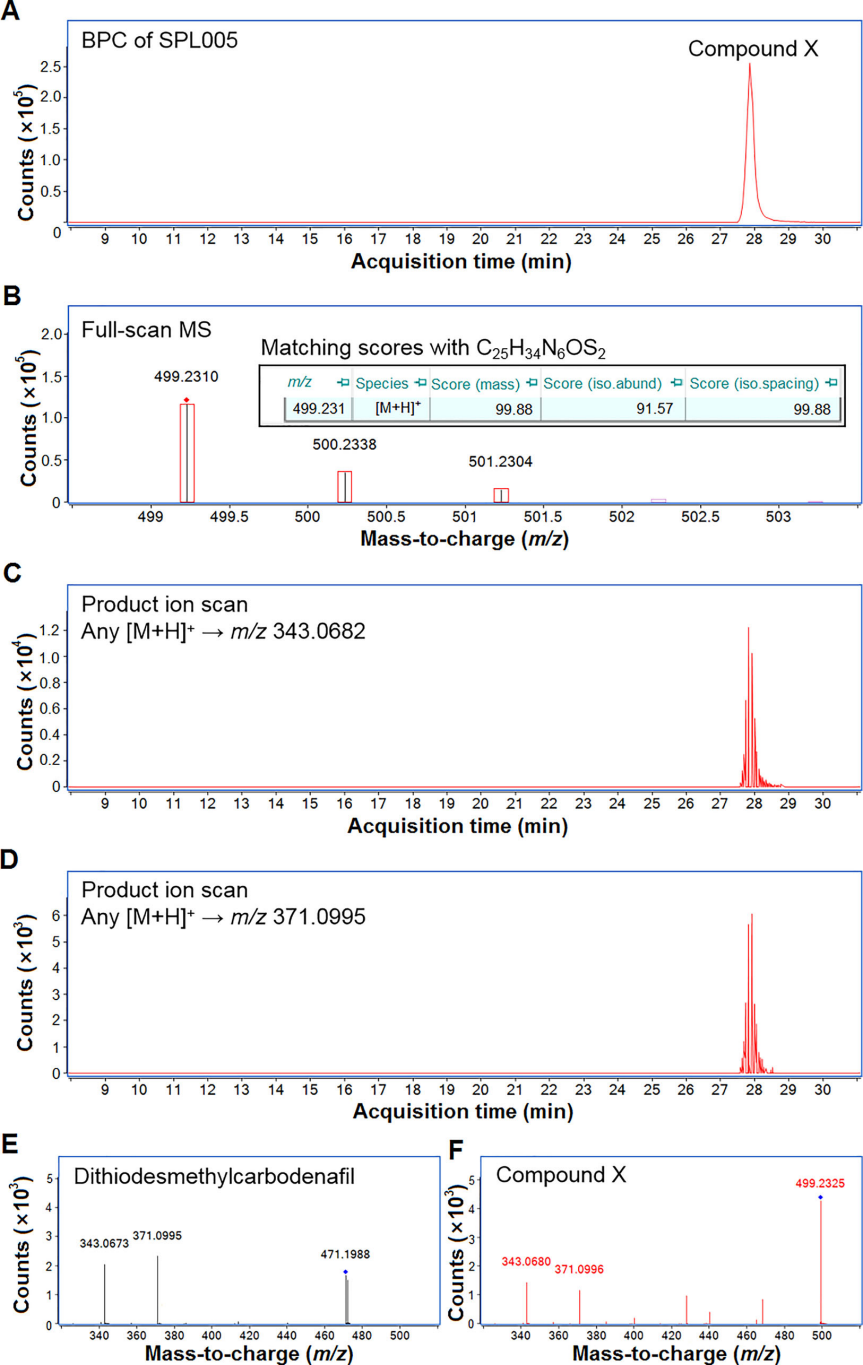
(A) Base peak chromatogram (BPC) of SPL005 with one unidentified peak, initially assigned as compound X; (B) full-scan mass spectrometry (MS) with a protonated molecule ([M + H]^+^) at *m/z* 499.2310 (also showing the matching scores of the observed mass, isotopic abundance distribution, and isotopic spacing of compound X with C_25_H_34_N_6_OS_2_); (C) and (D) product ion scan employing the non-targeted screening at *m/z* 343.0682 and *m/z* 371.0995; (E) and (F) common fragmentation patterns shared by dithiodesmethylcarbodenafil and compound X at averaged collision energies (CEs).

SPL005 was initially flagged for suspected adulteration through PDE5 inhibition assay, where it showed to inhibit the PDE5 enzyme [15]. An LC-QTOF-MS analysis [16] later unveiled the presence of one unidentified peak, initially assigned as compound X at 27.85 min of the base peak chromatogram (BPC) (Figure 1A). The full-scan MS in Figure 1B shows a protonated molecule ([M + H]^+^) at *m/z* 499.2310, suggesting a chemical formula of C_25_H_34_N_6_OS_2_ with a mass error of 0.40 ppm. The matching scores of the observed mass, isotopic abundance distribution, and isotopic spacing for compound X were also ascertained to be >80%. The suspected-target screening [16] matched the [M + H]^+^ precursor ion with two suspected analytes, i.e. 3,5-dimethylpiperazinyl-dithiodesmethylcarbodenafil and dithiopropylcarbodenafil, *via* the PCDL library.

**Figure 2.**
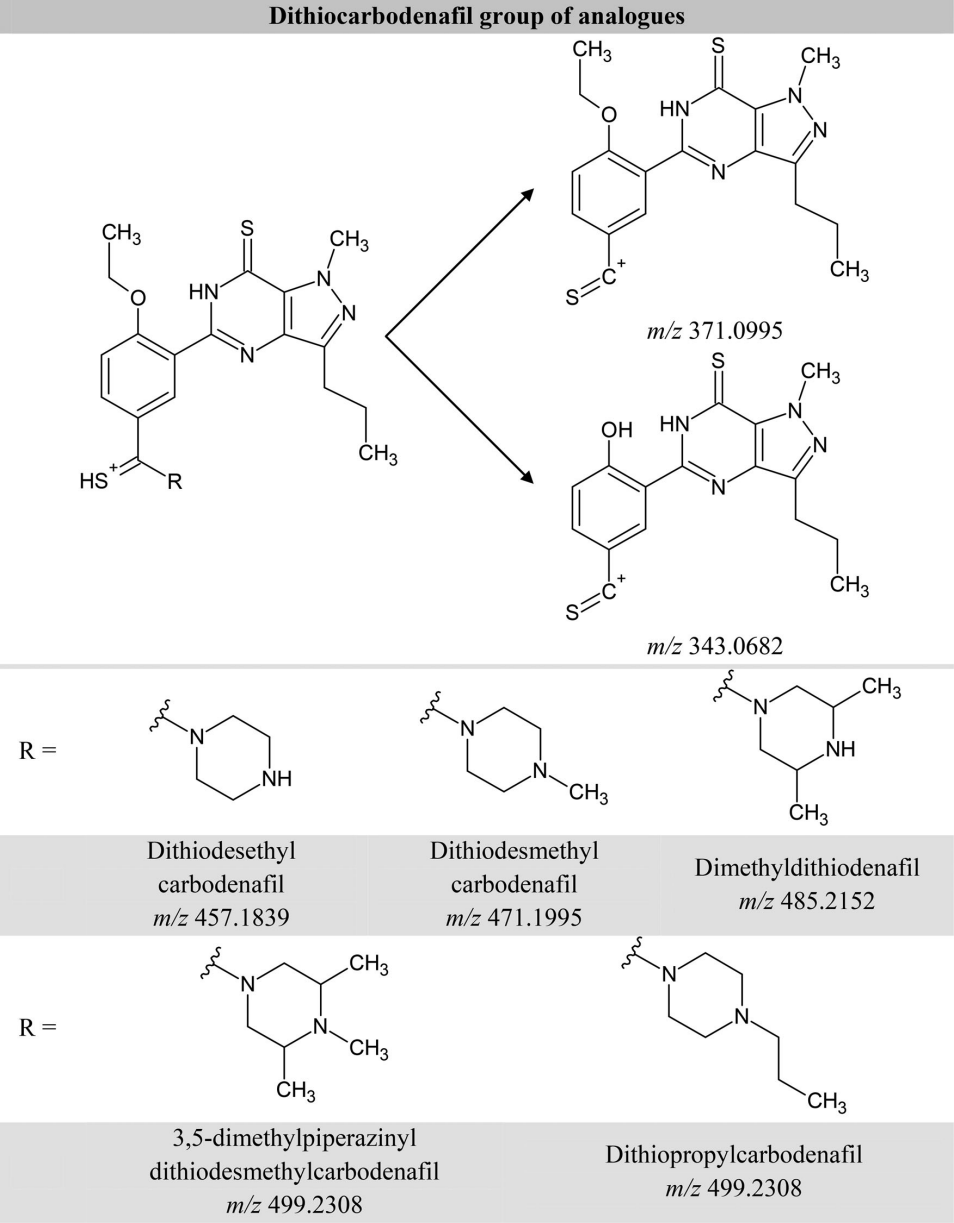
Proposed common fragmentation patterns shared by dithiocarbodenafil group of analogues.

In addition to this, the product ion scan employing the non-targeted screening [16] revealed the presence of two product ions corresponding to the common fragmentation patterns shared by the dithiocarbodenafil group of analogues, presented in Figure 2 due to the cleavage of piperazine ring and subsequent loss of ethene from the ethoxyphenyl moiety. Figure 1C and D further display the product ions’ signals at *m/z* 343.0682 and *m/z* 371.0995, which aligned with the peak of compound X within ±20 ppm mass error. Dithiodesmethylcarbodenafil CRM, which represents the dithiocarbodenafil group of analogues, shared the same common fragmentation patterns as compound X at averaged collision energies, shown in Figure 1E and F.

These findings indicated that compound X belongs to the dithiocarbodenafil group of analogues. However, both of the suspected analytes are structural isomers of one another. Besides, four other possible structural isomers could be generated based on these findings. Complementary technique such as LC-UV and NMR spectroscopy would, therefore, be highly valuable following analyte isolation and purification.

### LC-DAD and LC-UV of compound X

**Figure 3.**
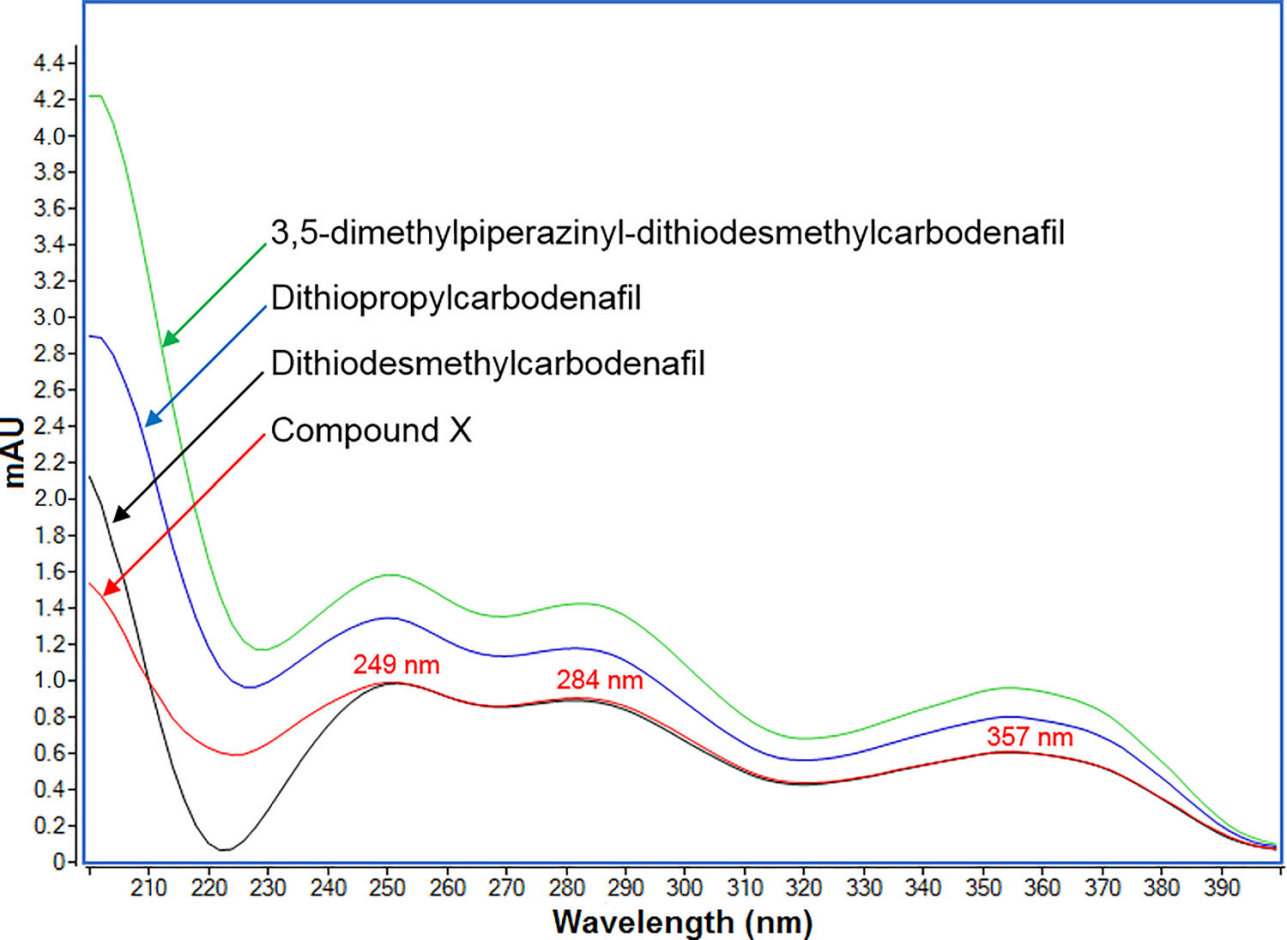
Overlaid ultraviolet (UV) spectra of three structurally related phosphodiesterase 5 (PDE5) inhibitor standards at 1 µg/mL and compound X isolated from SPL005.

Compound X (2 mg) in the form of pale-yellow solid was isolated from the LC-DAD and then analysed by employing LC-UV and NMR spectroscopy. Figure 3 displays the UV spectrum of compound X with maximum absorbance at 249, 284, and 357 nm, similar to that of dithiodesmethylcarbodenafil. The 3,5-dimethylpiperazinyl-dithiodesmethylcarbodenafil and dithiopropylcarbodenafil also exhibited similar UV spectrum patterns, overlaid as a comparison.

### NMR spectroscopy of compound X

Table 1 compiles the ^1^H and ^13^C NMR data of compound X in comparison with the structurally related PDE5 inhibitors, i.e. dithiodesmethylcarbodenafil [18], 3,5-dimethylpiperazinyl-dithiodesmethylcarbodenafil [19], and dithiopropylcarbodenafil [20]. The ^1^H and ^13^C NMR signal assignments of compound X, as well as 3,5-dimethylpiperazinyl-dithiodesmethylcarbodenafil and dithiopropylcarbodenafil, were comparable to that of dithiodesmethylcarbodenafil, except for the piperazine ring environment at positions 24–31. Indeed, all PDE5 inhibitors within the dithiocarbodenafil group of analogues possess similar skeletal configurations at positions 1–23, except for the different substitutes on the piperazine ring. Compound X was, therefore, characterised based on this skeletal structure.

**Table 1.** ^1^H and ^13^C NMR data (δ in ppm, *J* in Hz) of compound X and structurally related PDE5 inhibitors.

	Dithiodesmethylcarbodenafil		3,5-dimethylpiperazinyl- dithiodesmethylcarbodenafil		Dithiopropylcarbodenafil		Compound X	
	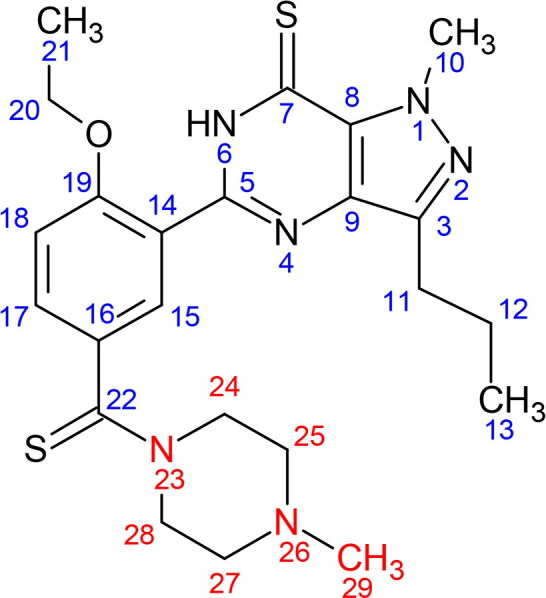		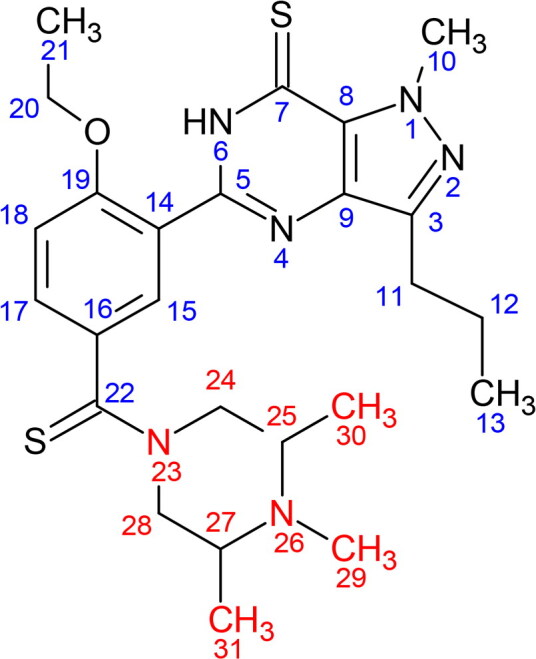		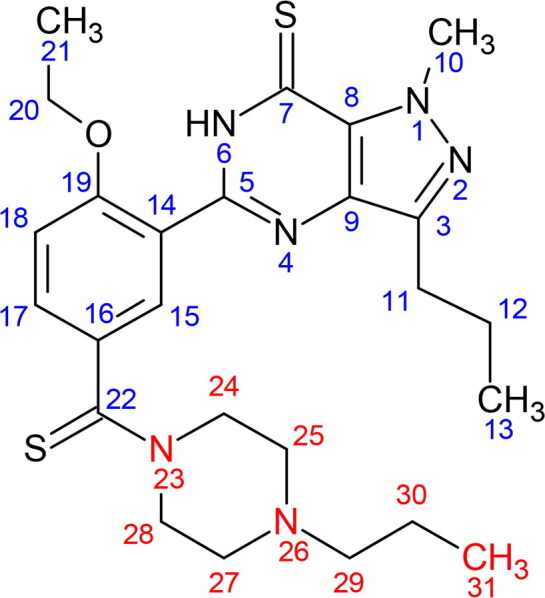		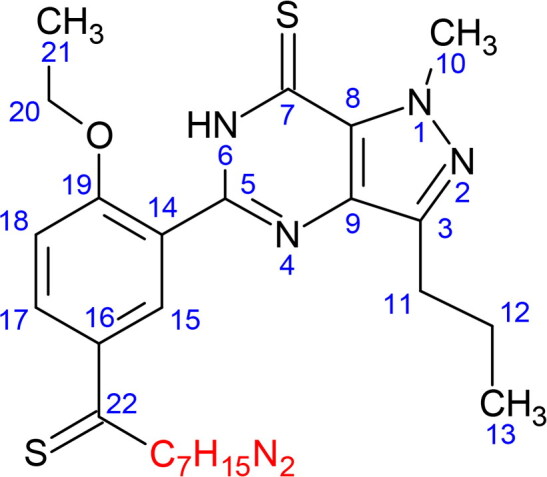	
	^1^H (δH)	^13^C (δC)	^1^H (δH)	^13^C (δC)	^1^H (δH)	^13^C (δC)	^1^H (δH)*	^13^C (δC)
1	NA	NA	NA	NA	NA	NA	NA	NA
2	NA	NA	NA	NA	NA	NA	NA	NA
3	NA	146.2	NA	144.7	NA	146.2	NA	144.6
4	NA	NA	NA	NA	NA	NA	NA	NA
5	NA	147.0	NA	148.2	NA	147.0	NA	146.7
6	12.59 (1H, s)	NA	13.30 (1H, s)	NA	12.61 (1H, brs)	NA	12.18 (s)	NA
7	NA	171.8	NA	171.6	NA	171.7	NA	170.2
8	NA	132.3	NA	131.8	NA	132.3	NA	132.2
9	NA	134.1	NA	133.6	NA	134.1	NA	133.7
10	4.52 (3H, s)	39.2	4.43 (3H, s)	39.5	4.53 (3H, s)	39.4	4.02 (s)	39.4
11	2.93 (2H, t, 7.5)	27.6	2.82 (2H, t, 7.0)	26.9	2.93 (2H, t, 7.5)	27.6	3.02 (t, 7.5)	26.2
12	1.87 (2H, sextet, 7.5)	22.3	1.75 (2H, sextet, 7.5)	22.0	1.86 (2H, sextet, 7.5)	22.3	1.89 (sextet, 6.9)	22.6
13	1.01 (3H, t, 7.5)	14.0	0.93 (3H, t, 7.5)	13.8	1.01 (3H, t, 7.4)	14.1	0.88 (t, 7.0)	14.1
14	NA	136.3	NA	134.6	NA	136.3	NA	137.7
15	8.41 (1H, d, 2.5)	128.1	7.70 (1H, d, 2.5)	128.2	8.42 (1H, d, 2.3)	128.1	8.51 (d, 2.5)	128.8
16	NA	118.5	NA	120.6	NA	118.4	NA	121.6
17	7.55 (1H, dd, 2.5, 8.0)	131.7	7.48 (1H, dd, 2.5, 8.0)	130.6	7.57 (1H, dd, 2.3, 8.6)	131.7	7.58 (dd, 1.2, 8.7)	131.8
18	7.06 (1H, d, 8.0)	113.0	7.19 (1H, d, 8.5)	112.6	7.07 (1H, d, 8.6)	113.0	7.09 (d, 8.7)	113.1
19	NA	156.9	NA	157.0	NA	156.9	NA	155.9
20	4.34 (2H, q, 7.0)	66.0	4.21 (2H, q, 7.0)	64.7	4.35 (2H, q, 7.0)	66.0	4.22 (q, 7.2)	65.1
21	1.69 (3H, t, 7.0)	14.8	1.38 (3H, t, 7.0)	14.4	1.70 (3H, t, 7.0)	14.8	1.42 (t, 6.9)	14.5
22	NA	199.3	NA	196.6	NA	198.8	NA	198.1
23	NA	NA	NA	NA	NA	NA	NA	NA
24	3.73 (2H, brs)	52.0	Ha 2.97 (1H, t, 12), He 5.20 (1H, d, 13)	55.1	3.71 (2H, brs)	52.3	3.60 (dd), 3.70 (dd)	51.7
25	2.50 (2H, brs)	55.2	2.31 (1H, m)	56.8	2.52 (2H, t, 4.7)	53.5	2.47 (brs)	53.7
26	NA	NA	NA	NA	NA	NA	NA	NA
27	2.68 (2H, brs)	54.3	2.21 (1H, m)	57.9	2.69 (2H, brs)	52.6	2.47 (brs)	53.4
28	4.48 (2H, brs)	49.5	Ha 3.15 (1H, t, 11.5), He 3.78 (1H, d, 15)	57.5	4.49 (2H, brs)	49.8	3.60 (dd), 3.70 (dd)	50.4
29	2.38 (3H, s)	45.5	2.20 (3H, s)	37.0	2.38 (2H, t, 7.5)	60.1	2.47 (brs)	36.8
30	NA	NA	1.12 (3H, d, 6)	17.7	1.54 (2H, sextet, 7.5)	20.0	1.05 (d, 7.3)	17.4
31	NA	NA	0.92 (3H, d, 6)	17.3	0.93 (3H, t, 7.4)	11.8	1.02 (d, 7.3)	17.4

Note: Positions 1–31 indicate either a hydrogen or carbon signal.

Abbreviations: s, singlet; brs, broad singlet; d, doublet; dd, doublet of doublet; t, triplet; q, quartet; m, multiplet; NA: not applicable.

*The number of protons for each ^1^H NMR signals cannot be established due to the presence of unknown impurities in the isolated compound X.

Supplementary Figure S1 shows the ^1^H NMR spectrum of the isolated compound X. A broad singlet peak at 2.47 ppm was assigned to a methyl group attached to a nitrogen atom at position H-29, based on the ^1^H NMR signal of dithiodesmethylcarbodenafil [18]. The chemically equivalent protons of the methine groups attached to the same nitrogen atom at positions H-25 and H-27 are predicted to have similar chemical shifts within the range of 2 to 3 ppm. Therefore, they were assigned at 2.47 ppm within the same broad singlet peak. Another chemically equivalent protons of the methylene groups at positions H-24 and H-28 are expected to produce higher chemical shifts due to the diamagnetic anisotropy effect from a nearby thiocarbonyl group compared to those of H-25 and H-27 [18]. Therefore, they were assigned at 3.60 and 3.70 ppm, taking into account the axial and equatorial protons [21]. Finally, the two methyl groups at positions H-30 and H-31 were assigned to two doublet peaks at 1.05 and 1.02 ppm, respectively. As well, the ^13^C NMR chemical shifts of compound X at positions C-29, C-30, and C-31 were similar to those of 3,5-dimethylpiperazinyl-dithiodesmethylcarbodenafil, indicating the presence of three methyl groups attached to the piperazine ring. These results ruled out the possibility of having an N-propylated linear chain group connected to the piperazine ring of compound X.

Isolation and structural elucidation of compound X through the LC-DAD and NMR spectroscopy are rather challenging due to the complexity of the ICP matrix, which typically contains multiple ingredients. Furthermore, the low quantity of the adulterant, often at trace levels relative to the matrix components, demands a larger sample size to isolate sufficient amounts of PDE5 inhibitor for different types of NMR experiments. Nevertheless, based on the obtained ^1^H and ^13^C NMR data, the identity of compound X could still be inferred as the signal assignments comparable to that of 3,5-dimethylpiperazinyl-dithiodesmethylcarbodenafil, particularly for the three methyl groups at positions 29–31 of the piperazine ring.

### Confirmation of compound X

**Figure 4.**
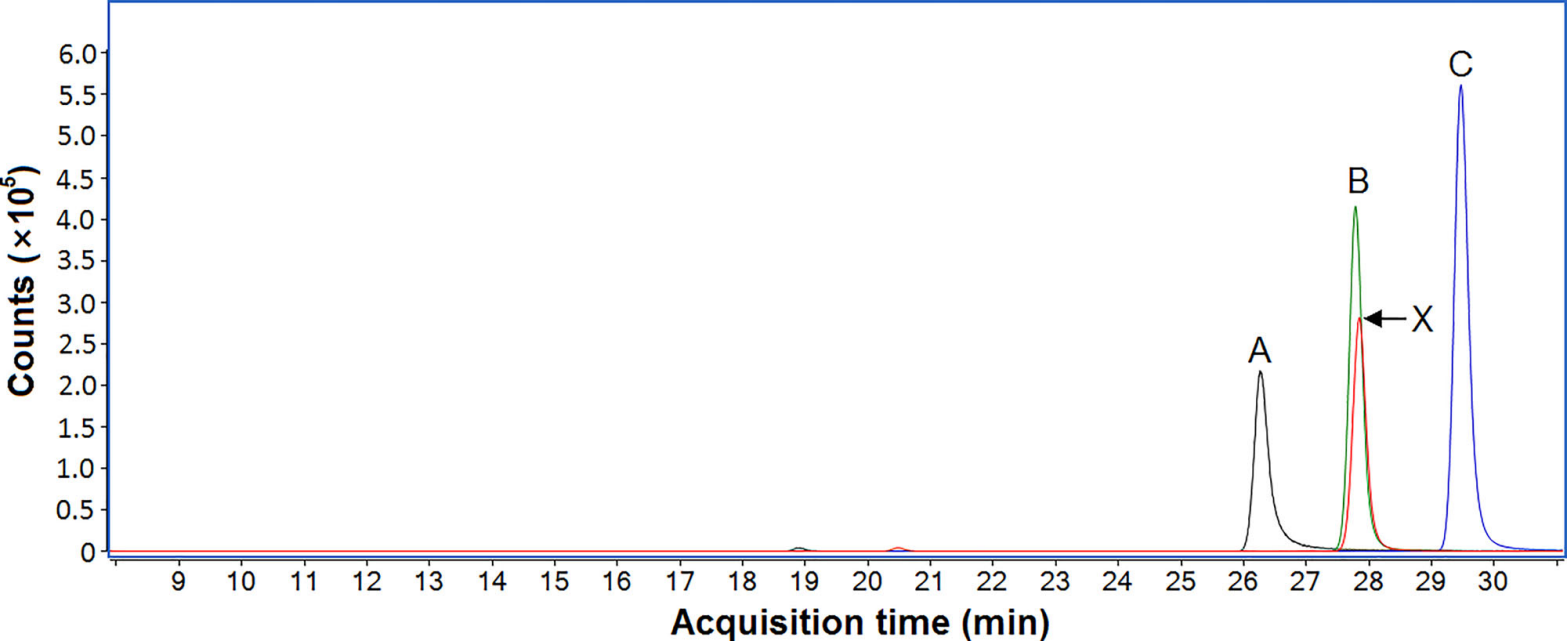
Overlaid base peak chromatograms (BPCs) of three structurally related PDE5 inhibitor standards at 1 µg/mL with (A) dithiodesmethylcarbodenafil, (B) 3,5-dimethylpiperazinyl-dithiodesmethylcarbodenafil, and (C) dithiopropylcarbodenafil; and (X) compound X isolated from SPL005.

The chemical structure of compound X was primarily elucidated from the LC-QTOF-MS, LC-UV, and NMR spectroscopy data. However, to confirm these findings, three CRMs of structurally related PDE5 inhibitors, i.e. dithiodesmethylcarbodenafil, 3,5-dimethylpiperazinyl-dithiodesmethylcarbodenafil, and dithiopropylcarbodenafil, were acquired to unambiguously conclude the identity of compound X, if possible, based on the chromatographic separation. Figure 4 shows an overlaid BPCs of the three structurally related PDE5 inhibitor standards at 1 µg/mL and compound X isolated from SPL005. Dithiodesmethylcarbodenafil eluted at 26.26 min, followed by 3,5-dimethylpiperazinyl-dithiodesmethylcarbodenafil and dithiopropylcarbodenafil at 27.78 min and 29.46 min, respectively. These findings indicated that the structural isomers were separated down to a baseline level, ensuring the specificity of each analyte.

Compound X, isolated from SPL005 eluted at 27.84 min, within ±0.25 min of the retention time of 3,5-dimethylpiperazinyl-dithiodesmethylcarbodenafil. Based on this finding, the 1 µg/mL standard solution of 3,5-dimethylpiperazinyl-dithiodesmethylcarbodenafil was spiked into the ICP sample solution at 1:10 v/v to substantiate the identity of compound X. The BPC of the spiked ICP showed only one peak at 27.80 min, similarly within ±0.25 min of the retention time of the CRM. These results, complemented by the previous data, concluded the identity of compound X as 3,5-dimethylpiperazinyl-dithiodesmethylcarbodenafil. The content of the adulterant was subsequently quantified at 8.4 mg per sachet of the ICP sample.

## Conclusion

This study’s comprehensive analytical procedure has identified an isomeric sildenafil analogue from an ICP marketed to enhance male sexual performance. Pre-screening with PDE5 inhibition assay and the following LC-QTOF-MS analysis revealed the presence of a suspected compound X. However, the suspected-target screening with an LC-QTOF-MS matched compound X with two suspected analytes that are structural isomers of one another. Compound X was, therefore, isolated from the ICP using an LC-DAD and then submitted to LC-UV and NMR spectroscopy analysis. The UV spectrum, as well as the NMR signals of compound X, closely matched to that of the 3,5-dimethylpiperazinyl-dithiodesmethylcarbodenafil. The identity of compound X was finally concluded by comparing its chromatographic separation with the structurally related PDE5 inhibitors. For the identification of structural isomers, baseline separation by way of chromatography is superior as their full spectral information are often indistinguishable, as demonstrated by 3,5-dimethylpiperazinyl-dithiodesmethylcarbodenafil and dithiopropylcarbodenafil. To our best knowledge, this is the first study to report an adulterated ICP containing 3,5-dimethylpiperazinyl-dithiodesmethylcarbodenafil.

## Supplementary Material

Supplemental Material
