# Supplementary material for: Isolation and identification of an isomeric sildenafil analogue as an adulterant in an instant coffee premix
Source: Forensic Sci Res. 2020 Nov 11;7(2):290–8. doi: 10.1080/20961790.2020.1829375 (PMC9245998; doi:10.1080/20961790.2020.1829375)
Supplement: Supplemental Material [file TFSR_A_1829375_SM2067.pdf]

Supplementary Data

Isolation and identification of an isomeric sildenafil analogue as an adulterant in an instant coffee premix

Ahmad Yusri Mohd Yusop[a,b], Linda Xiao[a], Shanlin Fu[a,*]

[a]*Centre for Forensic Science, University of Technology Sydney, Ultimo, NSW, 2007 Australia*

[b]*Pharmacy Enforcement Division, Ministry of Health, Petaling Jaya, Selangor, 46200 Malaysia*

*Corresponding author

Shanlin Fu, Centre for Forensic Science, University of Technology Sydney, Ultimo, NSW, 2007 Australia

Email: shanlin.fu@uts.edu.au

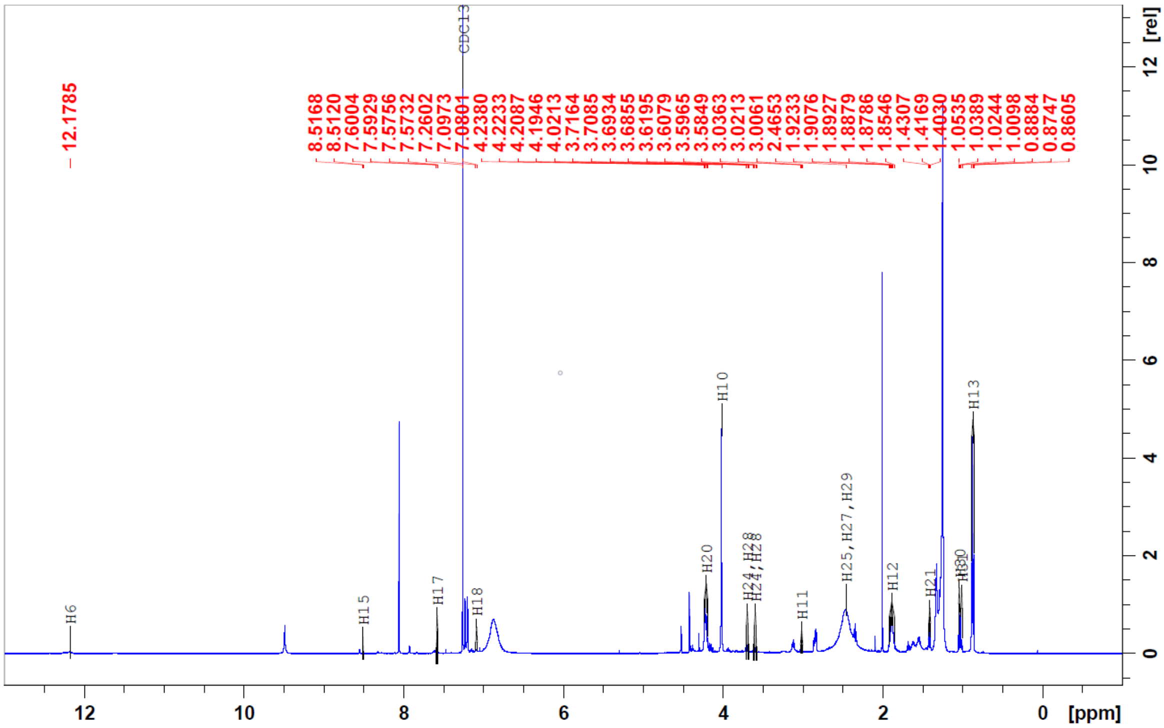

Figure S1: ^{1}H NMR spectrum of isolated compound X.
